# Is nodal biopsy a pre-requisite to surgery in biopsy proven pulmonary adenocarcinoma in the era of PET-CT?

**DOI:** 10.1186/1749-8090-10-S1-A256

**Published:** 2015-12-16

**Authors:** Naveed Abbas, Aurelie Fabre, David Healy

**Affiliations:** 1 Department of Cardiothoracic Surgery, St Vincent's University Hospital, Elm Park, Dublin, Republic of Ireland

## Background/Introduction

Lung cancer management is defined by stage and accurate pre-surgical staging is fundamental. The PET scan has become central to this process, but the technology has imperfect sensitivity and specificity. In determining nodal disease in early adenocarcinoma there appears to be a particular dilemma. The different subtypes of adenocarcinoma as per IASLC/ATS/ERS histopathological classification do not behave similarly in PET CTs, leading to increased dependence on staging EBUS or the "gold standard" mediastinoscopy.

## Aims/Objectives

We attempt to answer the question whether nodal biopsy is a pre-requisite to surgery in biopsy proven pulmonary adenocarcinoma.

## Method

Retrospective data from all patients undergoing surgical resection of adenocarcinoma with pre-operative staging limiting nodal disease to N1 and tumor size < T3 during the period of Jan 2012 to December 2014 was included. The data taken was from CT, PET and histopathology. Surgical decision was made after an MDM.

## Results

A total of 40 patients were included. The most common subtype of adenocarcinoma was acinar (AA) 42.5%, lepidic (LA) 20%, papillary (PA) 17.5%, micropapillary (MPA) and solid (SA) 10% each. PET was positive for nodal disease in 4 (10%) patients, while histology revealed nodal disease in 10 patients (25%). 2 PET positive patients were false positives. Nodal disease was most common in PA and AA subtypes. None of LAs had any nodal spread. 9/10 patients had a tumor size ≤ 25 mm. 13 patients (32.5%) had lymphatic invasion, of which 7 had established nodal spread. SUV max had a positive association with nodal disease above 5.0. SUV max below 5.0 was also associated with a smaller size.

## Discussion/Conclusion

There is a direct relationship between low SUVmax and small tumor size. PET CT scan has a low sensitivity in diagnosing nodal disease in early adenocarcinomas. The risk of nodal spread however is low in lepidic adenocarcinoma. We recommend that biopsy proven lepidic adenocarcinomas with a SUVmax below 5.0 on PET CT should be good candidates for surgical intervention without mediastinoscopy. In other adenocarcinoma subtypes, nodal disease suspected by size criteria on CT may merit sampling prior to resection.

